# Supplementary material for: The Pathogenic Role of Expanded CD8⁺CD28null Angiogenic T Cells in ANCA-Associated Vasculitis
Source: Biomedicines. 2024 Dec 26;13(1):26. doi: 10.3390/biomedicines13010026 (PMC11760873; doi:10.3390/biomedicines13010026)
Supplement: Supplementary file 1 [file biomedicines-13-00026-s001.zip › biomedicines-3291922-supplementary.pdf]

Supplementary Information

The Pathogenic Role of Expanded CD8+CD28null Angiogenic T Cells in ANCA-Associated Vasculitis

Haomiao Shen [1,†], Jinlin Miao [1,†], Haoyang Sun [1], Kui Zhang [1], Renli Liu [1], Zichao Li [2], Leyang Zhang [2], Peiyan Zhang [1], Jiawei Wang [1], Bei Zhang [1], Longyu Chen [1], Zhaohui Zheng [1,*], Ping Zhu [1,*]

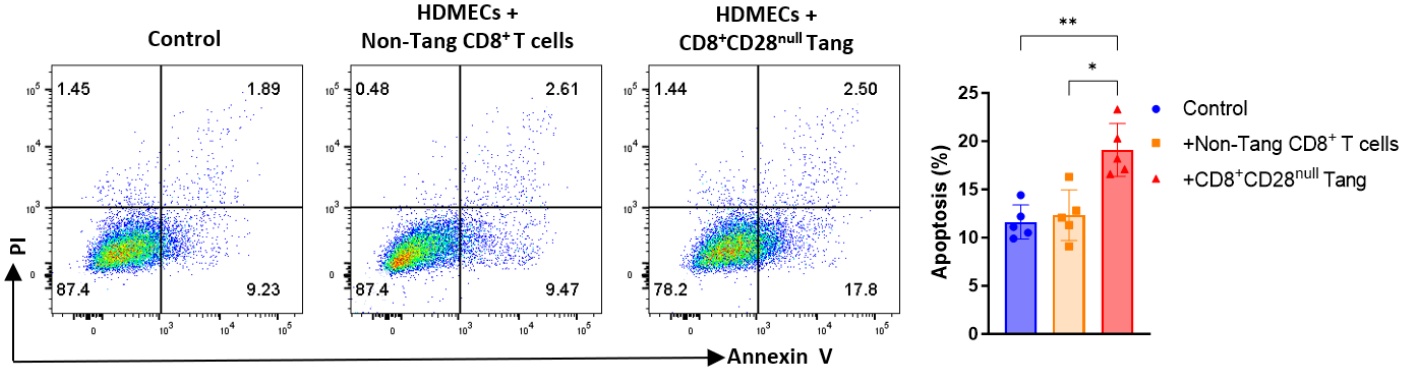

Figure S1. Induction of apoptosis in HDMECs by CD8$^+$CD28^{null} Tang from AAV patients.

Annexin V/PI staining was used to evaluate human dermal microvascular endothelial cells (HDMECs) apoptosis after 24 hours of direct co-culture with CD8$^+$CD28^{null} Tang and non-Tang CD8$^+$ T cells from AAV patients. HDMECs cultured alone were used as the control group. Significance levels are represented as follows: * $p < 0.05$; ** $p < 0.01$.
